# Microcins in *Enterobacteriaceae*: Peptide Antimicrobials in the Eco-Active Intestinal Chemosphere

**DOI:** 10.3389/fmicb.2019.02261

**Published:** 2019-10-09

**Authors:** Fernando Baquero, Val F. Lanza, Maria-Rosario Baquero, Rosa del Campo, Daniel A. Bravo-Vázquez

**Affiliations:** ^1^Department of Microbiology, Ramón y Cajal University Hospital, Ramón y Cajal Institute for Health Research (IRYCIS), Madrid, Spain; ^2^Bioinformatics Unit, Ramón y Cajal University Hospital, Ramón y Cajal Institute for Health Research (IRYCIS), Madrid, Spain; ^3^Department of Microbiology, Alfonso X El Sabio University, Villanueva de la Cañada, Spain

**Keywords:** microcins, chemosphere, colicins, bacteriocins, molecular ecology, *Enterobacteriaceae*, competition

## Abstract

Microcins are low-molecular-weight, ribosomally produced, highly stable, bacterial-inhibitory molecules involved in competitive, and amensalistic interactions between *Enterobacteriaceae* in the intestine. These interactions take place in a highly complex chemical landscape, the intestinal eco-active chemosphere, composed of chemical substances that positively or negatively influence bacterial growth, including those originated from nutrient uptake, and those produced by the action of the human or animal host and the intestinal microbiome. The contribution of bacteria results from their effect on the host generated molecules, on food and digested food, and organic substances from microbial origin, including from bacterial degradation. Here, we comprehensively review the main chemical substances present in the human intestinal chemosphere, particularly of those having inhibitory effects on microorganisms. With this background, and focusing on *Enterobacteriaceae*, the most relevant human pathogens from the intestinal microbiota, the microcin’s history and classification, mechanisms of action, and mechanisms involved in microcin’s immunity (in microcin producers) and resistance (non-producers) are reviewed. Products from the chemosphere likely modulate the ecological effects of microcin activity. Several cross-resistance mechanisms are shared by microcins, colicins, bacteriophages, and some conventional antibiotics, which are expected to produce cross-effects. Double-microcin-producing strains (such as microcins MccM and MccH47) have been successfully used for decades in the control of pathogenic gut organisms. Microcins are associated with successful gut colonization, facilitating translocation and invasion, leading to bacteremia, and urinary tract infections. In fact, *Escherichia coli* strains from the more invasive phylogroups (e.g., B2) are frequently microcinogenic. A publicly accessible APD3 database http://aps.unmc.edu/AP/ shows particular genes encoding microcins in 34.1% of *E*. *coli* strains (mostly MccV, MccM, MccH47, and MccI47), and much less in *Shigella* and *Salmonella* (<2%). Some 4.65% of *Klebsiella pneumoniae* are microcinogenic (mostly with MccE492), and even less in *Enterobacter* or *Citrobacter* (mostly MccS). The high frequency and variety of microcins in some *Enterobacteriaceae* indicate key ecological functions, a notion supported by their dominance in the intestinal microbiota of biosynthetic gene clusters involved in the synthesis of post-translationally modified peptide microcins.

## Introduction

The intestinal tract of mammals is a highly complex environment. It is an open environment partly influenced by factors external to the host, including food, swallowed environmental microorganisms (including those from the intestines of the same or other mammals), and abiotic environmental features. Consequently, the external microbial environment is “represented” in the intestine and can be considered an “invironment,” a shared space where the interior and the exterior of the organism merge (Baquero, 2012). The biotic part of the intestinal environment is essentially endowed by the functions of the host in the upper intestine, but the environment of the distal ileum and colonic space is dominated by highly diverse gut microbiota composed of trillions of microbes (Lozupone et al., 2012) with associations between themselves and the host in complex interactive networks. These networks have been refined during a long coevolutionary trajectory (Ley et al., 2008a, b), starting probably in vertebrates (525 million years ago), and refined later in mammals (200 million years ago); thus, our current intestinal microbiota can almost be considered another human organ (Baquero and Nombela, 2012). The intestinal chemosphere, the ensemble of chemical molecules in the lumen and on the surfaces of the gut, is particularly relevant to understanding the regulatory ecology of the microbiome. In this review, an important group of peptide-derived effectors of bacterial origin, the microcins, are examined in detail. However, it is critical to understand that the ecological effects of these molecules are necessarily modulated by a complex constellation of other chemicals, the chemosphere, influencing the composition, physiology, and the resilience of the microbiota.

Until recently, a conservative, reductive view of understanding the biological phenomena has favored the concept that a single (or few) molecular or biological entity is sufficient to explain the variation in frequency of particular bacterial populations in the individual host. Given this single-entity approach has been shown to be untrue, it should be presented with a more integrative perspective (Baquero, 2015).

In this review, we summarize basic knowledge to help the reader understand the role of a group of ribosomally synthesized, low-molecular-weight peptidic molecules with antimicrobial effects, the microcins, which influence bacterial interactions. Our aim is to suggest that the ecological activity of microcins should be understood within a much larger frame of ecological influences exerted by many other chemical compounds in the intestine, the intestinal eco-active chemosphere. We use the term “eco-active” to clarify that we restricted our interest to chemicals that play a role as factors of the local microbial ecology; i.e., bacterial growth-promoting molecules, growth-inhibiting (or killer) substances, and chemicals influencing bacterial genetic variation, genetic regulation, bacterial interactions, and colonization efficiency.

## The Intestinal Chemosphere: Molecular Ecology

The term “molecular ecology” had been proposed by one of the discoverers of microcins, the biochemist Carlos Asensio, as early as 1975 (Asensio, 1976). He posited it was “necessary to change the focus of the biochemist’s outlook on nature; a change of mood and style,” focusing more on a new attitude in search of ecological perspectives at a molecular level. This visionary approach was based on his early experiences, shared with one of the authors of this review (FB), concerning the first description of microcins, and low-molecular-weight antimicrobial agents produced by gut enterobacteria.

### The Intestinal Chemosphere and the Molecular Ecology of the Gut

The concept of a chemosphere at the core of the earliest studies on intestinal microorganisms, such as the work of Powers and Levine (1937), is also implicit in much later studies (Russell et al., 2013; Lee and Hase, 2014; Donia and Fischbach, 2015; Milshteyn et al., 2018). The microbiota works in a molecular chemosphere to which the microbiota itself contributes. In this review, we focus only on the fraction of the chemosphere comprised of natural chemical compounds present in the intestinal lumen (particularly in the large intestine), and not those that are part of or are tightly bound to the bacterial or host surfaces. The chemosphere is frequently subject to natural fluctuations, which are both the cause and the consequence of changes in the microbial community. Until very recently, the chemical environment of the gut has remained poorly defined, and therefore the “Molecular Ecology” of the environment where the microbiota was functioning had been poorly accessible. In recent years, the development of metabolomic approaches (mostly using proton nuclear magnetic resonance and mass spectrometry) has contributed to this field, attempting to define a human “fecal metabolome,” comprised of small molecules from digested food, mainly metabolites (and residues from metabolites) of human origin, and more importantly from the effects of microbiota acting on human, food, or microbial organic substances, or resulting from bacterial degradation (Matsumoto et al., 2012; Xu et al., 2015; Milshteyn et al., 2018). Multiomics approaches in combination with metabolic modeling will soon contribute to a more complete view of chemical flows in the intestinal microbiota (Sieow et al., 2019).

The number of detectable metabolites in the gut is vast. All these chemical substances might, by themselves or in combination, serve as molecular mediators of microbe-microbe, and microbe-host interactions (Lustri et al., 2017). An advancing field of research, following earlier studies on microbiota and intestinal nutrients (Hooper et al., 2002) is focusing on the “metabobiome,” that is, the network structure linking the composition of the intestinal microbiota and the intestinal metabolome (Xu et al., 2015). The gut microbiota has a considerable effect on the profile of mammalian blood metabolites (Wikoff et al., 2009). Given there is a core microbiota established in many (or most) human individuals (Turnbaugh et al., 2009), there should also be an accessory microbiota only present in distinct groups of individuals (Saric et al., 2007). Correspondingly, there should be a core and an accessory microbiota-derived metabolome (Xu et al., 2015). Part of the “species barriers” in transmission of bacteria among heterogeneous hosts (such as humans and food animals) could be due to discordances in the metabolic chemosphere of the intestine required for bacterial colonization in each type of host (Baquero, 2018; Nagpal et al., 2018). Bioinformatic approaches that are focused on the detection of secondary metabolites from the microbiota could be critical to casting light on this issue (Weber and Kim, 2016; Ozdemir et al., 2018). The variety of microbial metabolites in the gut is currently being explored by bioinformatic methods, such as ClusterFinder, to detect the biosynthetic gene clusters encoded in the genomes of the human microbiome (Donia and Fischbach, 2015). Gene clusters involved in the production of known oligosaccharide and ribosomally synthesized, post-translationally modified peptides (including microcins) were frequently identified, and this number could be further increased, given many biosynthetic clusters remain uncharacterized.

In any case, the abundance and complexity of the known microbiota-generated metabolites are overwhelming. Only the sub-metabolome of amine- and phenol-containing metabolites in fecal samples might comprise over 5000 different molecules (Xu et al., 2015). Compounds derived from dietary polyphenols, including chlorogenic acids, tannins, and flavonoids play an important role in the ecology of the intestinal microbiota (Popa et al., 2015). Carbohydrates, lipids, and proteins acquired from food or excreted by the host into the gut and eventually degraded or modified by the microbiota are also part of the chemosphere.

Mucins are particularly important as substrates for bacterial activity (Tailford et al., 2015; Corfield, 2018). In the neonatal period, human milk contains hundreds of glycans, including mucins, glycosaminoglycans, glycoproteins, and particularly human milk oligosaccharides, influencing the composition of the microbiota, mainly by modulating bacterial binding to intestinal surfaces, as in the case of *Escherichia coli* (Newburg and Morelli, 2014). Lipids in the milk, mostly free fatty acids, also have a role in microbiota construction. In infants, and also in adults, a number of bacterial gut populations have the ability to forage on glycans provided by the mucus layer covering the surface of the gastrointestinal tract, and are eventually released in the lumen by cell detachment. As a consequence, α- and β- linked N-acetyl-galactosamine, galactose, and N-acetyl-glucosamine can be incorporated into the chemosphere. Mucin glycans probably play a key role in selecting microbial communities along and across the gastrointestinal tract (Kashyap et al., 2013a, b; Tailford et al., 2015).

Dietary fiber- or host-derived (such as epithelial mucus) glycans produce many metabolites and can degrade into short-chain fatty acids such as acetate, butyrate, and propionate. This degradation requires a consortium of microorganisms linked by a trophic chain (Turroni et al., 2008). Other short-chain fatty acids, such as isobutyric, valeric, 2-/3-methylbutyric, caproic, and isocaproic are derived from amino acid metabolism. Phosphatidylethanolamine, derived from membrane lipids from animal hosts and bacteria, is degraded to glycerol and ethanolamine. Ethanolamine is a significant nutrient for gut microorganisms (Garsin, 2010; Kaval et al., 2018), as are probably phosphoinositides, sphingolipids, cholesterol, and eicosanoids (Bäckhed and Crawford, 2010). Bacterial action on dietary phospholipids (phosphoglycerides) such as choline, carnitine, or lecithin (phosphatidyl choline) gives rise to trimethylamine-N-oxide, acting as an osmolyte, assuring bacterial cell wall replication under stress and counteracting the effect of urea (Mukherjee et al., 2005; Lee and Hase, 2014).

Amino acids are actively produced by intestinal bacteria as electron acceptors in a highly anaerobic environment, frequently used together with reductive amino acid metabolites, such as phenylpropionic acid, and phenylacetic acid (Donia and Fischbach, 2015). Indole, a tryptophan metabolite, serves as a signaling molecule in bacterial interactions. It is from aliphatic amino acids, such as arginine, proline, and ornithine, that δ-aminovaleric acid is produced; threonine or methionine are the source of α-aminobutyric acid.

Proteins are present in vast amounts in the intestinal chemosphere. A gene catalog database of the human gut microbiome indicates the presence of nearly 10 million proteins; however, most of them are clearly intracellular proteins that are only available after bacterial lysis (Zhang et al., 2016). Proteins from the microbiota and the host are the target of metaproteomics (Xiong et al., 2015). From the approximately 6000 proteins that have been detected in the gut by metaproteomics, some two-thirds of them are of microbial origin (Verberkmoes et al., 2009; Erickson et al., 2012). More recent studies have identified more than 100,000 unique peptides associated with the microbiota (Cheng et al., 2017). The diversity of proteins is enhanced by post-translational modifications (by hydroxylation, methylation, citrullination, acetylation, phosphorylation, methyl-thiolation, S-nitrosylation, and nitration); in *E*. *coli* more than 5000 post-translational modification events been identified (Olsen and Mann, 2013). As in the metabolome, there is apparently a “core proteome” consisting of core functional categories (Verberkmoes et al., 2009). The intestinal proteome differs in the various intestinal regions, where variation in the local microbiota influences protein abundance and diversity (Lichtman et al., 2016).

In fact, there should be, at least in the colonic space, a wealth of molecules released by lysed bacteria (cell debris), including not only intracytoplasmic small molecules, nucleic acids, and proteins (many likely of ribosomal origin), but more importantly bacterial membranes releasing lipopolysaccharides (glycolipids), lipoproteins, phospholipids, and peptidoglycan fragments, resulting from lysis of bacterial cell envelopes. It has been estimated that approximately one-third of bacteria in the gut are dead organisms (Ben-Amor et al., 2005). However, the contribution of “bacterial waste” to the intestinal chemosphere remains scarcely investigated.

The microbiota influences the intestinal chemosphere by altering the production and/or consumption of simple chemical molecules such as water, oxygen, hydrogen, nitrogen, carbon monoxide, carbon dioxide, hydrogen peroxide, nitrogen oxide, sulfates, ammonium, methane, and ethylene, and metals serving as nutrients or cofactors. In particular, microbiota and oxygen balance in the gut are deeply linked (Vacca, 2017). Given most of the microbiota is composed of strict anaerobic organisms, when oxygen availability increases, as occurs during antibiotic therapy, these populations are reduced, favoring facultative-aerobic organisms, such as *Enterobacteriaceae* (Rivera-Chávez et al., 2017). Finally, among the metals, iron is widely considered as a nutrient for microbiota. In fact, it is one of the main chemicals involved in biological competition, including competition among bacterial populations and also with the host (Kortman et al., 2014). Changes in gut iron availability alters the microbiota, frequently favoring rapid-growing bacteria, such as some intestinal pathogens (Jaeggi et al., 2015).

### The Intestinal Chemosphere as a Field of Microbial Interactions

**FIGURE 1:**
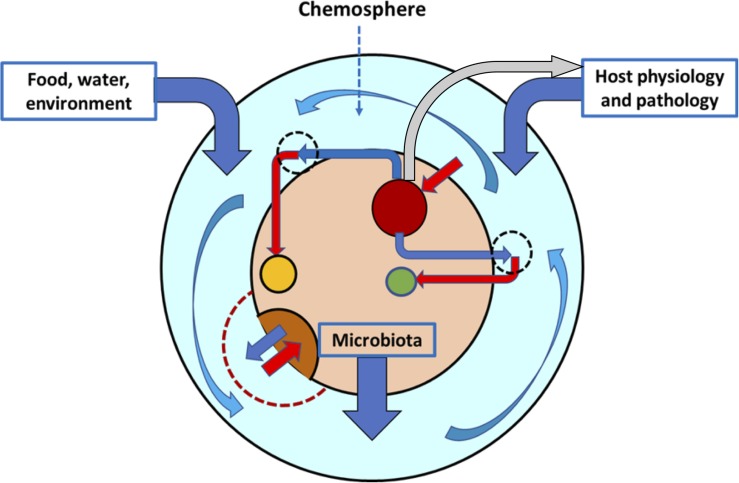
The intestinal chemosphere. The chemosphere (light blue layer) is the ensemble of chemical molecules of dietary and environmental origin, released (dark blue vertical arrows) by the physiological or pathological functions of the host or by the complex bacterial communities colonizing the intestine. The chemosphere surrounds the gut microbiota (inner beige circle), composed of a variety of bacterial populations (dark red, brown, and yellow circles). These populations contribute to the chemosphere with chemicals (dark blue angled arrows) that result, probably in combination with other local chemicals, in the growth or inhibition of the same or other populations (red arrows). The chemosphere might have local differentiations with sets of chemicals (hatched circles), but an important part of it is flowing (curved blue arrows). Some components of the microbiota can regulate the secretion of chemicals by the host (gray arrow).

Reciprocal interactions are probably the most frequent processes in microbial gut ecology and are deeply influenced by the intestinal chemosphere. The microbiota determines an important part of the chemosphere, and the chemosphere constitutes the common chemical environment of the microbiota (Figure 1). A long and common evolutionary history of microbial organisms within their chemospheres has refined and stabilized intermicrobial interactions so they produce reciprocal effects on the interacting partners. In fact, the microbiota not only provides chemicals to the chemosphere, but some of these compounds, such as tryptamine, can activate the epithelial protein-coupled receptor to increase colonic secretion, probably for the benefit of some populations (Bhattarai et al., 2018). The microbe-driven modification of the intestinal chemosphere in the intestine could be a major factor influencing pathogen restriction, a topic thus far insufficiently investigated (Rangan and Hang, 2017).

Competition dominates species and clonal interactions in the bacterial world (Foster and Bell, 2012; Stubbendieck and Straight, 2016), and most probably the diversity and stability of the intestinal microbiota depends on competitive interactions (Coyte et al., 2015). Given the local coexistence of populations competing in the intestinal chemosphere for the same energy sources, nutrients, and attachment surfaces, the multiplication of the fittest reduces the reproductive possibilities of the competitor (exploitative competition) (Hibbing et al., 2010). However, this antagonistic interaction rarely produces the extinction of the competitor. Environmental fluctuation/variation might favor this competitor in other (sometimes immediate) circumstances. Also, because punctual competition could be for the mutual benefit of “being together” (coexistence mutualism) in the presence of certain substrates or conditions, so that the maintenance of both earlier contenders is assured faced with a third competitor negatively influencing both. This concept has been presented as a case similar to the game “rock-paper-scissors” (Czárán et al., 2002). In addition, and as was mentioned earlier, collective-cooperative trophic actions involving various bacterial populations are required to degrade particular compounds of the chemosphere, such as glycans from dietary fiber or epithelial mucus.

Later in this review we analyze in some detail the fact that the intestinal microbial ecology includes a wealth of interactions in which the growth of some bacterial populations is inhibited by others, either more efficient in the competition for limited vital resources, or excreting toxic substances which are released in the chemosphere, which is termed interference competition (Hibbing et al., 2010). At first sight, these types of effects can be considered an indirect, coincidental, or unintentional allelopathy. However, it is difficult to decide whether the spectrum of allelochemicals (such as metabolites) released by particular groups or ensembles of groups in the microbiota has evolved to maintain a healthy species diversity based on negative interactions (Abrudan et al., 2012).

Directed allelopathy or amensalism, in which one bacterial population inhibits the growth of or kills another one in a non-reciprocal manner, likely resulting in a benefit for the offender, is much more specific in shaping microbial ecology than interference competition based in fight for nutrients. Amensalism might facilitate long-term genome evolution, given the DNA released from killed cells can be incorporated into the aggressor’s genome (García-Bayona and Comstock, 2018). The involved allelochemicals are considered “antibacterial compounds,” frequently “secondary metabolites” produced in the late stages of growth or during a stationary phase. It is of note that antimicrobials produced by bacteria cannot necessarily be considered (in our anthropocentric view) as weapons against “others,” but more as signaling agents (Linares et al., 2006; Chikindas et al., 2018), probably acting along gradients. In fact, the maintenance of species diversity requires a non-extinction outcome, even in amensalistic interactions. In this respect, it should be debated whether allelopathic substances have evolved as an attack or defense strategy. Experimental results have suggested that antibiotic production does not improve the ability of producers to invade a population of sensitive cells (Wiener, 1996). On the contrary, established colicin-producing populations in structured habitats, which allow the achievement of a critical (high) population density, can overcome potential susceptible competitors (Chao and Levin, 1981; Durrett and Levin, 1997). This view is supported by the frequent production of allelopathic substances in populations with slow growth or during a stationary phase, such as when they reach high density in structured habitats. Whether allelopathy is triggered by different types of stress is an interesting possibility to consider; certainly, competition occurs more frequently under limiting conditions. However, the production of allelotoxic compounds might require investing energy in costly biosynthetic processes, whereas an alternative immunity/resistance-based “defensive strategy” could be evolutionarily preferred (García-Gutiérrez et al., 2019). This view suggests that the protection of population borders is critical, probably preventing niche invasions that can provoke local extinctions. Finally, the antibacterial activity of some allelopathic agents is highly dependent on the surrounding chemosphere; for instance, on the available carbon sources, trypsin, or carbon dioxide (García-Bayona and Comstock, 2018).

### Microbial Growth Inhibitors in the Chemosphere

The ecological effects of intestinal chemical substances influence many dimensions. One aspect is their inhibitory effects on bacterial growth. In fact, intestinal microbiota can be considered a potential source of novel antimicrobials (Mousa et al., 2017). In the context of this review, we focus on metabolites and compounds arising from the action of intestinal bacteria that might inhibit bacterial replication or reduce microbial viability. As noted above, when defining the intestinal chemosphere, we are considering the chemical growth inhibitors that are available in the lumen, not those that are dependent on bacterial surfaces, such as those mediating contact inhibition (Willett et al., 2015; Chen et al., 2018).

#### Dietary Polyphenols and Carbohydrate Metabolites as Bacterial Growth Inhibitors

A number of phytochemicals, particularly polyphenols, are frequently bacterial growth inhibitors. In particular, flavonoids have direct antibacterial activity, eventually potentiating the effect of other antimicrobials (Cushnie and Lamb, 2011). It has been proposed that dietary flavonoids such as quercetin might even protect against some pathogens (Popa et al., 2015). Chlorogenic acids are extremely frequent in nature (approximately 400 have been reported), and among them, acyl-quinic acids are the most studied (Clifford et al., 2017). Chlorogenic acids have antimicrobial effects, and given these promote the increase in permeability of the outer and plasma bacterial membranes, they might also increase the effects of potentially active substances inhibiting microorganisms excluded by these barriers (Lou et al., 2011). Dietary fiber and polyphenols are metabolized to short-chain fatty acids and phenolic acids by the colonic microbiota (Russell and Duthie, 2011).

Short chain fatty acids resulting from the effect of microbiota on carbohydrates and polyphenols, mostly derived from the diet, are fermented by the gut microbiota and are in turn important effectors of microbial growth restriction. This inhibition occurs both by lowering pH, and by a pH-independent antibacterial effect. *Firmicutes* species mainly produce butyrate, whereas *Bacteroidetes* primarily produce acetate and propionate (den Besten et al., 2013; Ridaura et al., 2013). In newborns, the early (human milk-promoted) overgrowth of acetate-producing *Bifidobacterium* prevents a dangerous massive colonization with opportunistic, mostly Gram-negative, pathogens (Underwood et al., 2015). This modulation of the microbiota by acetate has inspired probiotic strategies using *Bifidobacterium* to correct metabolic disorders (Fukuda et al., 2011; Aoki et al., 2017).

Organic salts such as lactate and citrate have a growth-inhibitory activity on some species of microbiota (Zhitnitsky et al., 2017). Part of this activity is simply due to the pH effect. In fact, pH has a significant role in determining the species composition of human colonic microbiota; mildly acidic pH restricts Gram-negative bacteria, including *Enterobacteriaceae* and *Bacteroidetes*, particularly in the presence of short-chain fatty acids (such as acetate), favoring the growth of low-pH-tolerant microorganisms (Duncan et al., 2009). However, citrate has antibacterial activity independent of the pH effect on organisms of the colonic microbiota, such as *Fusobacterium* (Nagaoka et al., 2010).

#### Bile Acid and Lipid Bacterial Growth Inhibitors

The gut microbiota deconjugates and subsequently metabolizes the primary bile acids, cholic and chenodeoxycholic acid (cholesterol derivatives), into secondary bile acids, including terpenoids (Donia and Fischbach, 2015); bile salt hydrolases are key enzymes in the process (Jones et al., 2008). Both conjugated and unconjugated bile salts have direct antimicrobial activity (Sannasiddappa et al., 2017) and indirect actions on microbiota by modulating innate immunity. Inhibition occurs particularly in the proximal intestine; thus, if bile excretion is prevented, it results in bacterial overgrowth in the gut (Hofmann and Eckmann, 2006). The direct effects of bile salts in several Gram-negative bacteria probably result from action on membranes, which is in part compensated by the induction of efflux pumps (Thanassi et al., 1997; Rosenberg et al., 2003). Inhibition of Gram-negatives results in an increase in the proportion of Gram-positive bacteria (Friedman et al., 2018).

Lipids such as fatty alcohols, free fatty acids, and monoglycerides of fatty acids have antibacterial effects both on Gram-positive and Gram-negative bacteria, most probably due to damage to the bacterial envelopes (Thormar and Hilmarsson, 2007; Bergsson et al., 2011). Part of the antibacterial activity of milk is due to lipids, mostly medium-chain saturated, and long-chain unsaturated fatty acids and their monoglycerides released by lipases in the gastrointestinal tract (Isaacs, 2001).

#### Protein- and Amino Acid-Derived Bacterial Growth Inhibitors

Polyaminated molecules, biogenic amines, and polyamines are small polycation molecules derived from aromatic or cationic amino acids by decarboxylation, a process that can be mediated by intestinal microorganisms. *Bacteroides* (most importantly *B*. *thetaiotaomicron*) and *Fusobacterium* are important producers; however, polyamines are also produced by a “consortia” of other bacteria exchanging metabolites and forming collective chemical pathways (Matsumoto and Benno, 2007; Sakanaka et al., 2016; Nakamura et al., 2018). Polyamines include spermidine, homospermidine, norspermidine, putrescine, cadaverine, and 1,3-diaminopropane. The rate of production and degradation of biogenic amines and polyamines has consequences in microbial ecology (Pugin et al., 2017; Tofalo et al., 2019). Polyamines such as putrescine, spermine, and spermidine are known to be antibacterials, effective against Gram-positive bacteria (Bachracht and Weinstein, 1970). However, these compounds can also alter bacterial membrane permeability in Gram-negatives and in fact can serve as potential scaffolds for new antibacterial agents (Blanchet et al., 2016). In addition, polyamines can act as regulators of bacteriocin production, thus indirectly influencing competitive bacterial interactions (Yi-Hsuan and Chen-Chung, 2006).

Secretory N-acyl homoserine lactones mediating bacterial *quorum-sensing* in bacterial populations might also have antibacterial activity (Pomini and Marsaioli, 2008; John et al., 2016; Saroj et al., 2017). Indole-based intercellular communication molecules might also have antibacterial effects, eventually enhancing the effect of antibiotics (Biswas et al., 2015). Recently, it has been shown that *quorum-sensing* molecules involved in interspecies cell-to-cell communication, such as the signal autoinducer-2 from *E*. *coli*, influences the species composition of gut microbiota (Bivar, 2018).

Intestinal unconjugated bilirubin, resulting from the catabolism of hemes (from senescent erythrocytes), has a weak antimicrobial activity against *E*. *coli* and *Klebsiella pneumoniae* (Terzi et al., 2016). Biliverdin might influence inflammatory mediators (Overhaus et al., 2006). Finally, non-ribosomal peptides of bacterial origin are rarely found to be associated with mammals’ microbiota (Donia and Fischbach, 2015).

#### Host Defense Antimicrobial Peptides and Proteins

Host defense antimicrobial molecules produced by epithelial cells of the intestine can be secreted into the intestinal lumen of mammals, such as antimicrobial peptides (α-defensins, β-defensins, and cathelicidins), protective carbohydrate-bonding proteins (C-type lectins), and RNAses (Gallo and Hooper, 2012; Meade and O’Farrelly, 2019). Secretion of these molecules is frequently controlled by bacterial-originated signals and might influence the composition of the microbial community (Salzman et al., 2010). However, they remain mostly attached to the inner and outer mucus layers of epithelium, embedded in the mucin glycoprotein layer (Meyer-Hoffert et al., 2008; Dupont et al., 2014). Given detached fragments of this mucin layer can serve as a source of bacterial nutrients, a possible effect of these antimicrobial peptides in the gut lumen, and not only on the epithelial surfaces, cannot be excluded. Finally, immunoglobulins, particularly secretory IgA, are produced by plasma cells and transported into the lumen through the intestinal epithelial cells but remain surface-attached to block epithelial receptors (Mantis et al., 2011). These large antimicrobial proteins also have effects on the microbiota composition, particularly on *Proteobacteria* (Mirpuri et al., 2014).

#### Bacteriocins

In 1925, André Gratia, a Belgian microbiologist, was the first to detect antagonisms between *Enterobacteriaceae* strains (Gratia, 1925), and this early work was followed by that of Pierre Fredericq (Fredericq and Levine, 1947), who identified colicins, the first bacteriocins. Bacteriocins, ribosomally synthesized proteins, are specific effectors of bacterial inhibition or death, and they are particularly active on phylogenetic relatives of the producer (Cotter et al., 2005, 2013). Bacteriocins are considered important modulators of intestinal microbiota (Lenski and Riley, 2002; Kirkup and Riley, 2004; Corr et al., 2007; Angelakis et al., 2013; Million et al., 2013). The classification of bacteriocins, a heterogeneous group of substances of various bacterial origins, is a highly debatable and even confusing issue (Chikindas et al., 2018), even though the various classification proposals do not differ in the essential (Klaenhammer, 1993; Van Belkum and Stiles, 2000; Kemperman et al., 2003; Heng and Tagg, 2006; Arnison et al., 2013; Balciunas et al., 2013; Alvarez-Sieiro et al., 2016; Dicks et al., 2018). Two clearly recognizable broad groups, are the long thermolabile peptides, including colicins, and a heterogeneous ensemble of small thermostable peptides, including the microcins. Lantibiotics (lanthionine and methyllanthionine containing peptides) are also thermostable. Note that thermostability is often correlated with resistance to proteolysis (Daniel et al., 1982), an important trait for stability in the intestinal environment. A systematic search of previously described bacteriocin molecules (*n* = 1360, 1000 of them from Gram-positives) has been performed on 317 genomes of the gut microbiota (Drissi et al., 2015). *Firmicutes* and *Bacteroidetes*, the most predominant phyla in the human microbiota, produce the largest number of bacteriocins. However, based on the chemical structure, the authors suggest that bacteriocins produced by *Proteobacteria*, which are rich in cationic charges and α-helices, might have higher antibacterial activity (Drissi et al., 2015).

## The Microcins

### Historical Background of the Discovery of Microcins

Microcins were originally defined as eco-active, low-molecular-weight excreted molecules (less than 10 kDa), such as amino acids and short ribosomally synthesized peptides produced by Gram-negative organisms, and presenting resistance to proteases, extreme pH, and high temperatures. Microcins were discovered in 1974 in the process of identify effectors of enterobacterial sequential displacements in the microbiota of newborns (Baquero and Asensio, 1976). Even though the number of species cumulatively increases with time in newborns, the absolute number of bacterial cells is extremely high from the early days. The microcin’s historical screening was performed in conditions resembling the high-density colonization of the colonic space, using minimal media to mimic possible nutritional deficiency, and was designed to detect only low-molecular-weight molecules, microcins (meaning small bacteriocins), which are able to pass and exert activity through cellophane membranes, excluding molecules over 10,000 Da. This screening excluded conventional bacteriocins, large proteins that could be degraded by intestinal proteases. Low-molecular-weight growth-inhibitory substances were consistently detected in *Enterobacteriaceae* from the human newborn intestine (15% of the tested strains). The first comprehensive review on microcins was in 1984 (Baquero and Moreno, 1984). At this time, these growth-inhibitory substances were identified as small peptides and occasionally as amino acids or amino acid-derived compounds. Other secondary metabolites or chemical substances of bacterial origin with low molecular weight and high antimicrobial activity are apparently very rare or are produced in small amounts. The possibility that microcins could be considered as potential drugs was considered from this early period, and in fact the microcins remain as promising, but not yet developed, antibacterial agents (Gillor et al., 2004; Zucca et al., 2011; Collin and Maxwell, 2019). Although the designation “microcins” currently refers mainly to ribosomally synthesized peptides, for the purposes of this review we would like to recapitulate the original “functional” meaning as small eco-active molecules mediating bacterial interactions in the gut. However, we should acknowledge that amino acids and amino acid derivatives with antimicrobial activity do not correspond in a strict sense to microcins as they are understood today, and they should in fact be considered as “historical microcins.”

### Historical Amino Acid or Amino Acid-Derived Microcins; Secreted Amino Acids as Inhibitors

In early studies focusing on low-molecular-weight inhibitors excreted by *Enterobacteriaceae*, several excreted amino acids and amino acid-derived compounds were included among microcins. This was the case for L-valine, which inhibits growth of *E*. *coli* strain K-12 due to repression of the aceto-hydroxybutyrate-forming system, leading to an inhibitory shortage of isoleucine. Also, methionine derivatives, such as microcin 15 m, inhibit the first enzyme of the methionine biosynthetic pathway, homoserine-O-trans-succinylase (Baquero et al., 1984). Early research on microcins (Baquero and Asensio, 1976) had indicated that among *Enterobacteriaceae* from the neonatal human intestine, 3% hyperexcreted L-valine, and 7% putative methionine derivatives (such as microcin 93 m), the inhibitory activity being reversed by adding methionine (10 mg/ml) (Aguilar et al., 1982). In other studies, *E*. *coli* excretion of D-alanine has been detected; in general, the bacterial excretion of D-amino acids might have bacterial growth-inhibitory activity. Either glycine or D-amino acids inhibit the growth of *E*. *coli* (Hishinuma et al., 1969), altering lipoprotein binding in the outer membrane (Tsuruoka et al., 1984), and triggering biofilm disassembly (Kolodkin-Gal et al., 2010). D-amino acids also induce in eukaryotic (intestinal?) cells the toxic formation of superoxides, and trigger apoptosis (Bardaweel et al., 2013). In particular, D-arginine is probably involved in microbial interactions and contributes to microecological diversity (Álvarez et al., 2018). Despite the possible importance of these findings, research on the physiology and ecological consequences of bacterial secretion of amino acids and amino acid derivatives in complex ecosystems remains very limited (Krämer, 1994), and only in recent years has it been systematically investigated (Aliashkevich et al., 2018).

### Microcins as Small Peptides

In more recent times, the term “microcins” has been applied essentially to small peptides secreted by microorganisms (mostly *Enterobacteriaceae*) that are able to inhibit other bacteria. Microcins are non-SOS-inducible, ribosomally synthesized peptides, in some cases only active after post-translational modification. In 1949, the Belgian microbiologist Pierre Fredericq named the antagonistic substance previously described by Gratia in 1925 as “colicin V.” The possibility that this low-molecular-weight molecule could be considered a microcin was acknowledged by Fredericq in a personal face-to-face *ad hoc* meeting in Liège with one of the authors of this review (FB). Low molecular weight was in fact instrumental to differentiating them from colicins in the microcin kick-off consensus conference at the Alhambra, Granada, Spain, in 1983, which was attended by top international experts in the field including Volkmar Braun, Roberto Kolter, Jordan Konisky, Claude Lazdunsky, Bauke Oudega, Anthony Pugsley, and Maxime Schwartz. Thirty years later, in 2013, a consensus on universal nomenclature of ribosomally synthesised and post-translationally modified peptides (RiPPs) was presented (Arnison et al., 2013). Most of these active RiPPs are initially synthesized as a long precursor peptide, typically ∼20–110 residues in length, encoded by a structural gene. The “core peptide” is the region that is transformed in the bioactive molecule. Microcins were recognized as a separate family among these natural products (Arnison et al., 2013).

#### The Functions of Microcins

Microcins had been discovered as molecules influencing interbacterial interactions in complex microbial ecosystems, regulating microbial communities; this basic ecological function is at the core of this review. In addition to this basic function (Chung and Raffatellu, 2019), other interactions are also influenced by microcin-producing organisms, involving not only the microbial community but also the human or animal hosting the microbiota.

Microcins might have functions involving interactions with eukaryotic host cells. An important aspect is if microcins can cross the intestinal-blood barrier to produce systemic effects on the host (Dicks et al., 2018). Some microcins, such as MccJ25, have interactions with integrins, eukaryotic transmembrane receptors involved in cells’ extracellular matrix adhesion and potentially regulating the cell cycle (Hegemann et al., 2014). In fact, the lasso peptide MccJ25 can act as a pro-apoptotic eukaryotic antimicrobial peptide, thus being potentially active in anticancer therapy (Soudy et al., 2017), as are other bacteriocins and microcins (Cornut et al., 2008). Strains producing microcins MccH and MccM, as the Nissle strain, tends to target neoplastic cells (Stritzker et al., 2007), and MccE492 has antitumorigenic properties (Lagos et al., 2009). It has been proposed that the decrease in potentially antineoplastic bacteriocins and microcins in healthy individuals might contribute to the initiation of non-advanced colorectal neoplasia; however, when neoplasia is advanced, a higher frequency of microcinogenic strains occurs (Kohoutova et al., 2014). Microcins are part of complex gene clusters, such as the colibactin gene cluster located in the genetic island KPHPI208 of *K. pneumoniae*, likely inducing some degree of host DNA damage (e.g., regulatory functions and genotoxicity) (Lai et al., 2014). Similarly, *E*. *coli* Nissle, 1917 harbors a gene cluster, the “pks island” allow production of colibactin, causing potential genotoxicity (Olier et al., 2012). In addition, some microcins, such as those producing accumulation of oxazole compounds derived from MccB17 and other thiazole/oxazole-modified microcin-producing bacterial strains, might significantly influence host immune responses, leading to intestinal inflammatory effects (eventually providing food for the microbe). In general, they have an immunoregulatory effect mediated by the glycoprotein CD1d-restricted pathways, thus influencing antigen-presentation functions (Iyer et al., 2018).

Finally, microcins and microcin-related molecules might also have regulatory functions inside the bacterial cell. MccC triggers the stringent response and persistence in both sensitive and producing cells (Piskunova et al., 2017). Microcins might have functions related to bacterial maintenance of mobile genetic elements, such as plasmids; thus, acting as a post-segregational killing mechanism; that is, bacteria losing a plasmid are penalized with cell death (Fedorec et al., 2019).

#### The Microcin Classification

Approximately 15 peptidic microcin molecules have been identified, but the chemical structure is only known for 8 of them. Peptidic microcins are currently grouped into 2 classes (Rebuffat, 2012). Class I peptidic microcins, such as microcins MccB17, MccC, MccD93, and MccJ25 are small (less than 5 kDa) plasmid-encoded peptides requiring extensive backbone post-translational modifications. The term “post-translational thiazole/oxazole-modified microcins” (TOMMs) has also been suggested for MccB17 related bacteriocins (Melby et al., 2014). Class II peptidic microcins (Duquesne et al., 2007b; Vassiliadis et al., 2011; Santos et al., 2017) are larger (5–10 kDa), and can be subdivided into class IIa, including the plasmid-mediated microcins MccL, MccV, and MccS, not requiring post-translational modifications and having, respectively 2, 1, or no disulfide bond(s); and class IIb, such as the chromosomally encoded microcins MccE492, MccM, and MccH47, and carrying (MccM, MccH47, and MccI47) or not a C-terminal post-translational modification, involving a catechol-siderophore moiety (Patzer et al., 2003; Vassiliadis et al., 2010).

#### Microcins’ Mechanisms of Action

Microcins are antibiotic peptides, blocking vital functions in the target cell. They act by forming pores in the bacterial membrane (MccV, MccE492, and MccL), inhibiting aspartyl-tRNA synthetase, essential in protein synthesis (MccC), inhibiting the DNA gyrase GyrB, resulting in double DNA breaks (MccB17). Some others block the secondary RNA polymerase channel, impairing transcription and acting on cytochromes to inhibit cellular respiration (MccJ25), impairing the cellular proton channel (MccH47 and probably MccM and MccI), or the ATP synthase (MccH47). Some modes of action have been studied in detail and others remain to be confirmed. Colicins, much larger polypeptides, mainly act by pore formation, nuclease activity (DNase, 16S rRNase, and tRNase activities), and blocking peptidoglycan synthesis (colicin M).

Antimicrobial production should be balanced with appropriate mechanisms of self-protection by the producing organisms (immunity). Among others, these mechanisms involve acetyltransferases (MccC), production of immunity proteins (Class IIb microcins), efflux pumps (MccB17, MccJ25, and ppGpp-regulated), or inhibition of DNA gyrase supercoiling activity (MccB17), which are detailed later.

Once the peptides or their derivatives with antibacterial activity are released from the producer cell, action on other bacterial cells requires uptake mechanisms. Uptake depends frequently on outer membrane receptors, mainly OmpF and OmpC, but also on receptors involved in iron uptake (FhuA, FepA, Cir, and Fiu). Several microcins use the “Trojan horse” strategy of mimicking essential nutrients (such as essential amino acids or iron-siderophores) to be incorporated into the cell. Frequently, failure in nutrient uptake mechanisms results in microcin resistance in non-producer organisms. Next, in a simplified manner, we will review the main modes of action of these antibiotic peptides.

##### Class I microcins

The Microcin B17 (MccB17) structural gene, *mcbA*, encodes a 69-amino acid inactive precursor that undergoes at least 2 steps of post-translational modification, leading to the formation of oxazole and thiazole rings, and resulting in the toxic MccB17 molecule. These steps are performed by the McbBCD enzyme complex in subsequent reactions of cyclization, dehydration, and dehydrogenation involving the dipeptides Gly-Ser (oxazole) and Gly-Cys (thiazole), present in the MccB17 unmodified precursor (Li et al., 1996). Modification of the tripeptides Gly-Ser-Cys and Gly-Cys-Ser leads to the formation of oxazole-thiazole and thiazole-oxazole, respectively. The discovery of the process of MccB17 peptide maturation has been instrumental for overall progress of the field of biological synthesis of oxazoles-thiazoles and, more importantly, of post-translational modification (Yorgey et al., 1994). The recent elucidation of the microcin B synthase octameric protein complex has been instrumental to understand the process of conversion of a ribosomally synthesized peptide in a DNA gyrase inhibitor, culminating 30 years of research (Ghilarov et al., 2019). Interestingly, compounds very similar to microcin B can target molecular machines other than gyrase. A full set of MccB17 homologous proteins was identified in the genome of *K. pneumoniae*; one of them, klebsazolicin from *K*. *pneumoniae* subsp. *ozenae* is targeting the 70S ribosome, obstructing the peptide exit tunnel, and overmapping with group B streptogramins (Metelev et al., 2017b).

Mature MccB17 is exported outside the cell by a specific ABC transporter (McbE–McbF). TldD/TldE is a protease that removes the leader peptide from the MccB17 precursor, allowing MccB17 export by the McbE–McbF transport system (Allali et al., 2002; Tsibulskaya et al., 2017). The leader peptide does not intervene in microcin activity. The lethal cellular target of MccB17 is the GyrB subunit of DNA gyrase (Vizan et al., 1991). Inhibition of GyrB results in an impairment of DNA replication in sensitive cells, producing an SOS response (Herrero et al., 1986). Alterations in DNA packaging by MccB17 might increase the bacterial mutation rate, as in the case of novobiocin, also targeting GyrB (Chang et al., 2003). Most of the studies of MccB17 have been performed with *E*. *coli* strains; however, there are reports of MccB17-like activities in environmental *Pseudomonas*, such as *P*. *syringae* and *P*. *antarctica* (Metelev et al., 2013; Lee et al., 2017), encoded with an almost identical genetic structures to that of MccB17.

Microcin J25 (MccJ25) is a 21-amino acid antimicrobial peptide with a lasso structure. The study of lasso peptides has generated increasing interest due to their high stability and possible bioengineering applications in the design of enzyme inhibitors or to antagonize receptors (Rosengren and Craik, 2009). Lasso peptides are a class of ribosomally synthesized peptides, with a unique three-dimensional structure produced by a lasso peptide synthetase, and the formation of a macrolactam ring (Rebuffat et al., 2004; Ducasse et al., 2012; Yan et al., 2012; Sumida et al., 2019). Microcin J25 is active against *Salmonella* species and *E*. *coli* (Lopez et al., 2007). To enter into target cells, MccJ25 uses the outer membrane protein FhuA, the receptor for ferrichrome (a hydroxamate siderophore) involved in iron uptake (Salomón and Farías, 1993). Klebsidin, an MccJ25-like lasso peptide from *Klebsiella*, likely has a species-specific short host range, given it is only internalized in *E*. *coli* when expressing the FhuA homolog from *Klebsiella pneumoniae* (Metelev et al., 2017a). Once MccJ25 reaches the periplasmic space, it interacts with the inner membrane protein SbmA to enter the cytoplasm (Salomón and Farías, 1995). MccJ25 inhibits at least 2 intracellular targets, the secondary channel of RNA polymerase (Adelman et al., 2004; Braffman et al., 2019), resulting in transcription impairment, and the cytochromes bd-I and bo3, leading to inhibition of cellular respiration (Galván et al., 2018).

Microcin C (MccC). In this review we use the designation MccC, but in the literature the acronym McC has been used, to avoid confusion with the *mccC* gene, encoding an microcin C transport protein. Microcin C is the smallest microcin known to date. In fact, it is the smallest peptide of ribosomal origin, encoded by the smallest *E*. *coli* gene, of only 21 bp (González-Pastor et al., 1994). It is built by only seven amino acids forming an N-formylated heptapeptide with covalently attached C-terminal adenosine monophosphate and a propylamine group attached to the phosphate (Guijarro et al., 1995). This phosphorus atom determines the chirality of the microcin, and might condition its antibacterial activity (Severinov et al., 2007). The length of the peptide is evolutionarily conserved, given larger peptides strongly reduce MccC production and activity (Zukher et al., 2019). In *Yersinia*, the peptide-cytidine antibiotic is activated inside the cell by the TldD/E protease, suggesting that proteolytic processing might optimize activity and reduce toxicity (Tsibulskaya et al., 2017). MccC enters the target cells by the porin OmpF in the outer membrane and is then guided through the inner membrane by YejABEF, an ABC transporter (Novikova et al., 2007). In fact, microcin uses the Trojan horse strategy to penetrate the cell, through N-acylphosphoramidate, deceiving target cells. Once inside, it becomes toxic after being processed (Metlitskaya et al., 2006). First, the microcin will undergo excision of the formyl group at the N-terminus; then, in a second step, the peptide domain will be removed, with the active molecule mimicking the aspartyl adenylate, acting as a strong inhibitor of aspartyl-tRNA synthetase and inhibiting protein synthesis at the translation step (Metlitskaya et al., 2009).

##### Class IIa microcins

Microcin V (MccV), previously named colicin V, is an 88-amino acid peptide encoded by the *cvaC* gene. It contains a disulphide bond in the C-terminal sequence that is formed during post-translational modification. MccV is secreted by *E*. *coli* through a specific exporter composed of the proteins CvaA, CvaB, and TolC; MccV is only bactericidal after it is exported (Zhang et al., 1995) and is active against related bacteria belonging to the genera *Escherichia*, *Klebsiella*, *Salmonella*, and *Shigella* (Håvarstein et al., 1994). MccV is recognized only by Cir, an outer membrane receptor for catecholate siderophores, and its uptake is dependent on the TonB complex, providing the necessary proton motive force (Chehade and Braun, 1988). Moreover, MccV activity also depends on the cytoplasmic membrane protein SdaC, also involved in serine uptake (Gérard et al., 2005). The activity of MccV is related to membrane channel formation and disruption of membrane potential (Yang and Konisky, 1984).

Microcin L (MccL) is a peptide produced by the strain *E*. *coli* LR05 that exhibits strong antibacterial activity against related *Enterobacteriaceae*, including the *Salmonella enterica* serovars Typhimurium and Enteritidis (Sablé et al., 2003). MccL uptake requires the outer membrane receptor Cir, similar to MccV. Moreover, like MccV activity, MccL activity depends on the inner membrane protein TonB that transduces the proton motive force to transport iron siderophore complexes across the outer membrane. The MccL target is probably the bacterial membrane. In a preliminary study, it had been observed that high levels of MccL disrupt the inner membrane potential of *E*. *coli* cells; however, no permeabilization of the membrane had been detected (Morin et al., 2011).

Microcin N (MccN) is active against *E*. *coli* and *S.* Typhimurium but not against *Listeria monocytogenes* or *Campylobacter jejuni* (Wooley et al., 1999). To date, its uptake and mechanism of action are unknown. MccN displays sequence similarities with the class IIb MccE492 (Lagos et al., 1999), but lacks the C-terminal region necessary for recognition by catecholate siderophore receptors. Thus, focusing on the sequence identities, it has been suggested that MccN could have a target similar to MccE492, and both probably need ManY/ManZ inner membrane proteins (Bieler et al., 2006).

Microcin S (MccS), like microcins MccM and MccH47, was discovered by investigating the reason for the successful effect of a probiotic extensively used in functional gastrointestinal disorders. In this case, it was produced by *E*. *coli* G3/10, a component of the probiotic drug Symbioflor 2. MccS is encoded in the megaplasmid pSYM1 and has a genetic organization similar to other class IIa microcins, and it is the largest of all known microcins (11.67 kDa). MccS is lethal to the virulent enterohemorrhagic and enteropathogenic *E*. *coli*, but the mechanism of action has not been elucidated with certainty (Zschüttig et al., 2012, 2015).

Microcin PDI (MccPDI). The term “PDI” is an acronym for “proximity-dependent inhibition,” given a close physical proximity between producer and susceptible strains is required. An MccPDI precursor protein (McpM) interacts with a conserved motif of the outer membrane porin OmpF on susceptible cells, ultimately resulting in lethal membrane damage (Eberhart et al., 2012; Lu et al., 2019). Other proximity-dependent (or contact-dependent) inhibition phenomena acting on stationary-phase bacteria have been described, sometimes secondary to the overproduction of bacterial glycogen; however, the mechanism of inhibition remains elusive (Lemonnier et al., 2007; Navarro Llorens et al., 2010).

##### Class IIb microcins

Microcin E492 (MccE492) was isolated for the first time from *K*. *pneumoniae* (de Lorenzo, 1984), and is active against closely related bacteria. The chromosomal genes needed for active microcin production were cloned in *E*. *coli* for heterologous expression and characterization (Wilkens et al., 1997). The presence of a serine-rich region located at the C-terminus was surprising. The precursor of microcin undergoes a post-translational modification before being secreted. In the glycosylation process, the C-terminal serine is bound through an O-glycosidic link to a linear trimer of N-2,3-(dihydroxybenzoyl)-L-serine (DHBS) (Thomas et al., 2004). DHBS is a catechol siderophore, similar to other siderophores such as enterobactin and salmochelin. These molecules bind to iron and import it into cells via high-affinity receptors so that the producer strains become more competitive when placed in an iron-poor environment. In addition, the siderophore-microcin complex binds ferric iron selectively through the catecholate receptor, and could work as a siderophore (Thomas et al., 2004). MccE492 recognizes FepA, Fiu, and/or Cir as receptors in the outer membrane. The main receptor is FepA (Strahsburger et al., 2005). It should be remembered that MccV is only recognized by the Cir catechol-siderophore receptor. Once more, the complex formed by the inner membrane proteins, TonB-ExbB-ExbD, uses the proton motive power from the cytoplasmic membrane to convey energy to the outer membrane, allowing microcin intake (Thomas et al., 2004). Although the serine-rich region at the C-terminus is important for recognition by catecholate-siderophore receptors, it is not required for the microcin activity (Bieler et al., 2006). Once in the periplasmic space, MccE492 interacts with the inner membrane proteins ManY/ManZ of the mannose permease and induces channel or pore formation, and TonB-dependent inner membrane depolarization, followed by cell death (Bieler et al., 2006). However, it is unknown whether microcin has other targets in the cytoplasm (Destoumieux-Garzón et al., 2006).

Microcin H47 (MccH47), microcin M (MccM), and probably microcin I (MccI47) belong, just as MccE472, to the catechol siderophore microcin group (Vassiliadis et al., 2010). Antimicrobial activities are restricted to some species of *Enterobacteriaceae*. These peptides have a serine-rich domain at the C-terminus that is necessary for recognition by the outer membrane receptors but not required for activity (Bieler et al., 2006). Catecholate receptors (FhuA, Cir, and Fiu) in *E*. *coli* and (IroN, Cir, and FepA) in *Salmonella*, are essential for recognizing the siderophore microcin (Patzer et al., 2003) and lead microcins to the periplasmic space. Mutations in catecholate receptors have been associated with microcin resistance (Vassiliadis et al., 2010). The antibiotic activity of MccE492 requires the integrity of mannose permease (ManX/ManY/ManZ), but this is not the case for MccH47 or MccM (Vassiliadis et al., 2011; Peduzzi and Vandervennet, unpublished data). Interestingly, post-translational modifications increase the antibacterial activity for all class IIb microcins by mimicking the natural siderophores.

The proton channel is the minimal structure necessary for ATP synthase and is sufficient for MccH47 antibiotic action (Rodríguez and Lavina, 2003). The target of MccH47 is the F0F1 ATP synthase, and particularly its F0 membrane element, which serves as a proton channel. To date, the mechanisms of action of MccM and MccI are not known, although it is suspected that they act in the same way as MccH47, impairing the cellular proton channel. MccV acts similarly, and in fact, it has been suggested that MccH47 is probably related to MccV (Azpiroz et al., 2001; Azpiroz and Laviña, 2007).

Microcin N (MccN) is also known as microcin 24 (O’Brien and Mahanty, 1994). The uptake of MccN is dependent on the presence of SemA and/or TonB. Both are genes that code for membrane proteins within *E*. *coli* and are involved in microcin resistance and sensitivity. The mechanism of action has not been elucidated, but MccN appears to have DNAse activity (O’Brien, 1996).

#### Mechanisms of Immunity and Mechanisms of Resistance to Microcins

Immunity to microcins should be clearly distinguished from microcin resistance. Immunity explains the absence of “self-killing” in producing strains (this has been previously described); however, resistance means acquired insusceptibility to external microcins. Resistance might complement immunity in producer strains; for instance, an excreted microcin might not be internalized again because of a “resistance” mutation in a porin. Most mechanisms of resistance to microcins involve mutations. The possibility of acquisition of microcin-inactivating enzymes by horizontal gene transfer has not yet been investigated but cannot be ruled out. Whether self-immunity mechanisms can be converted into acquired-resistance mechanisms is a possibility, as occurs with antibiotics (Benveniste and Davies, 1973). Certainly, mutational resistance might evolve in microcin-susceptible bacteria during amensalistic-competitive interactions. A number of resistance mutations might have significant biological costs for the bacterial cell (e.g., reducing permeability) or specific uptake mechanisms (e.g., siderophores).

Specific immunity proteins and/or non-specific resistance proteins are required for the viability of the microcin producer bacteria (Kolter and Moreno, 1992). Most importantly, immunity proteins expel microcins using ATP-binding cassette transporters (Beis and Rebuffat, 2019). Immunity genes are typically encoded in the same operon, close to the genes involved in microcin production, such as structural genes, post-translational modification genes, and secretion genes (Baquero and Moreno, 1984). The elucidation of the microcin immunity protein structure will cast some light on the mechanisms of immunity, just as with bacteriocin immunity systems in lactic acid bacteria (Klaenhammer, 1993; Bastos et al., 2015).

The evolution of microcin production in combination with specific self-protection immunity mechanisms remains uncertain, but it is an attractive field of basic evolutionary research. In the following section, immunity and resistance to microcins are considered according to the 2 major microcin groups (class I and II) (Gaillard-Gendron et al., 2000; Pons et al., 2002).

##### Immunity and resistance to class I microcins

Immunity to several microcins in this group, including MccB17, MccC, MccJ25, and MccD93, involves the presence of efflux pumps. Concerning specific immunity to microcin B17, the expression of 3 genes, present in the microcin gene cluster, is required. These genes, *mcbE*, *mcbF*, and *mcbG* encode the 3 proteins McbE, McbF, and McbG, respectively. McbE and McbF constitute the microcin export system; their activity is needed for resistance to MccB17. McbG is a pentapeptide protein that protects cells that synthesize MccB17 from its own action, blocking the inhibition of DNA gyrase (Garrido et al., 1988). It is only when these 3 genes are expressed that cells are fully immune to their own toxic peptide. If one of the 3 genes is repressed, partial immunity phenotypes are shown. Whether the McbG mechanism of immunity contributes to the protection of fluoroquinolones in bacteria producing MccB17 is an interesting possibility. The widespread target-protection Qnr proteins involved in plasmid-mediated resistance to fluoroquinolones belong to the pentapeptide repeat family and share sequence homology with McbG (Tran and Jacoby, 2002; Rodríguez-Martínez et al., 2011). The first studies indicated that a plasmid carrying the entire MccB17 operon or a vector that expresses only the *mcbG* gene produces a 2–8× decrease in sensitivity to quinolones (Lomovskaya et al., 1996). However, the expression of Qnr does not produce resistance to MccB17 (Jacoby et al., 2015).

In *Escherichia coli*, resistance to external MccB17 occurs by mutations in OmpF, the outer membrane porin F, in the inner membrane SbmA transporter protein, and in the target of antimicrobial action, GyrB (tryptophan at position 751 is replaced by arginine). This mutational change in topoisomerase does not influence the susceptibility to coumarins or quinolones (del Castillo et al., 2001; Mathavan and Beis, 2012).

Regarding Microcin C, self-immunity of producing strains requires an efflux pump, and also number of enzymes able to detoxify MccC. This is the case of the MccE acetyltransferase, which is also protective against a number of toxic aminoacyl-nucleotides (Agarwal et al., 2011). MccE is homologous to the chromosomally encoded acetyltransferase, RimL, acting on L12 ribosome proteins, which also provide MccC and albomycin (a hydroxamate-type siderophore antibiotic) resistance (Novikova et al., 2010; Kazakov et al., 2014). In addition, a serine carboxypeptidase MccF protects against MccC (Agarwal et al., 2012). The carboxypeptidase MccF is similar to *E*. *coli* LdcA, acting on cell murotetrapeptides (Tikhonov et al., 2010).

Resistance to external MccC in non-producers occurs by mutations in YejABEF, an ABC transporter, preventing the uptake of the compound (Novikova et al., 2007). Orthologs of some of these MccC detoxifying enzymes might occur in non-MccC-producing bacteria, which could be protected (resistance) from the action of this microcin (Nocek et al., 2012). As probably strains producing MccC have a strong effect on intestinal competitors, the possibility of a flow of MccC detoxifying enzymes by horizontal gene transfer cannot be excluded.

Immunity to microcin J25 involves the protein McjD, ensuring highly specific export of MccJ25 and self-immunity to the peptide (Clarke and Campopiano, 2007; Gu et al., 2015; Husada et al., 2018; Romano et al., 2018) and possibly YojI (Vincent and Morero, 2009). They are efflux pumps that require TolC to extract the microcin from the producing bacteria (Delgado et al., 1999). Resistance to MccJ25 in *E*. *coli*-sensitive strains involves alterations in the outer membrane receptor FhuA (a siderophore receptor, explaining cross-resistance with albomycin, and a sideromycin) and the inner membrane proteins TonB, ExbB, ExbD, and SbmA. Given microcin J25 inhibits *E*. *coli* RNA polymerase, mutations in RpoB and RpoC are associated with resistance (Yuzenkova et al., 2002).

##### Immunity and resistance to class II microcins

Self-immunity for class II microcins involves membrane-associated small peptides, ranging from 51 to 144 amino acids, which protects the producing strain from its own antibacterial product in a highly specific way. Thus far, the 3-dimensional structure of these peptides has not been elucidated.

The protein involved in immunity to microcin V, MccV (formerly colicin V), has a molecular weight of approximately 6.5 kDa (Frick et al., 1981). The genetic determinant of MccV immunity protein, *cvi*, is located in a 700-base-pair fragment downstream from the region involved in its production (Gilson et al., 1987). The expression of the *cvi* gene was assessed under conditions of iron excess or depletion and immunoblots have shown that production of the immunity protein Cvi is iron dependent. The *cvi* promoter was located approximately 50 bp upstream from the *cvi* structural gene and was associated with a previously identified Fur binding site. The *cvi* promoter is also consistently inducible by iron depletion, and like other genes, encodes a transporter accessory protein, *cvaA* (Boyer and Tai, 1998). Resistance to MccV in non-producing cells has been analyzed in *E*. *coli* by transposon mutagenesis. Mutants in the *sdaC* (also called *dcrA*) gene, which is involved in serine uptake and is required for C1 phage adsorption, eliminate the bactericidal activity of this microcin (Gérard et al., 2005). Mutations in OmpF porin also result in MccV resistance (Jeanteur et al., 1994).

Immunity to MccL in producing organisms involves the *mclL* immunity gene, which was identified upstream of the *mclC* structural gene, and encodes a 51-amino acid protein with 2 potential transmembrane domains (Sablé et al., 2003; Pons et al., 2004). Resistance to MccL results from deficient uptake mediated by the outer membrane receptor Cir (colicin I receptor). Moreover, MccL bactericidal activity has been shown to depend on the TonB protein that transduces the proton motive force of the cytoplasmic membrane to transport iron-siderophore complexes across the outer membrane (Morin et al., 2011).

Immunity to microcins MccE492, MccH47, MccM, and MccI47 in the producer strains is provided by the inner membrane proteins, MceB, MchB, McmI, and MchS3, highly conserved in class IIb microcins, containing a putative transmembrane region (Duquesne et al., 2007a). The gene *mceB*, which encodes a protein of 95 amino acids, has been found in the strain *K*. *pneumoniae* RYC492. The gene *mchB* has been found in the microcin producer strains *E*. *coli* H47, *E*. *coli* CA46, *E*. *coli* CA58, and *E*. *coli* Nissle, 1917, and in all cases confers self-immunity to MccH47. The gene *mcmI*, has been found in the strains *E*. *coli* CA46, *E*. *coli* CA58, and *E*. *coli* Nissle, 1917, and confers self-immunity to microcin M. The strain *E*. *coli* H47 contains a truncated *mcmI* gene version that is not functional. The gene *mch*S3 has been found in the strains *E*. *coli* H47, *E*. *coli* CA46, and *E*. *coli* CA58, although the structural gene of MccI47, *mchS2*, is only present in the strains *E*. *coli* H47 and *E*. *coli* CA46 (Laviña et al., 1990; Patzer et al., 2003; Poey et al., 2006; Vassiliadis et al., 2010).

Microcin E492 immunity is negatively regulated by MceF (Tello Reyes, 2006). The gene *mceF* shows many similarities to the gene *mcmM*, which encodes a protein of 228 amino acids and 7 transmembrane domains and is present in the strains *E*. *coli* CA46, *E*. *coli* CA58, and *E*. *coli* Nissle, 1917. No evidence has been found that *mcmM* is necessary for immunity to MccM or MccH47 (Bravo-Vázquez, Doctoral Thesis). A sequence encoding for a 156-amino acid protein with 3 transmembrane domains, McmT, presumptively associated with MccH47 and MccM immunity, was cloned from *E*. *coli* Nissle, 1917, the producer strain. The plasmid containing the *mcmT* gene provided resistance in the recipient strain to MccH47 and MccM, and partial resistance to MccE492 and MccV (Bravo-Vázquez, Doctoral Thesis). A homologous MccT protein was found in *E*. *coli* O157:H7, the same region also containing the gene *mchA*, encoding a glycosyl transferase, essential in the biosynthetic pathway of MccE492, MccH47, MccM, and MccI47 (Bravo-Vázquez, Doctoral Thesis).

Mutations in three *E*. *coli* K12 genes, *tonB*, *exbB*, and *semA*, reduce sensitivity to MccE492 in non-producing strains; *tonB* and *exbB* genes had previously been shown to be involved in the uptake of siderophore (Pugsley et al., 1986).

Immunity to MccS depends on the gene *mcsI* encoding a 216-amino acid protein of the CAAX amino terminal protease protein family (Zschüttig et al., 2012).

Immunity to MccPDI involves a protein (McpI) that forms a multimeric cytoplasmic complex with itself; however, the detailed mechanisms remain unknown (Lu et al., 2019). Non-producer resistant *E*. *coli* strains display a mutation in a critical amino acid residue involved in the interaction of MccPDI with the outer membrane porin F (OmpF). Resistance mutations are present not only in this protein (or in OmpR), but also in AtpA, AtpF (ATP synthase), DsbA, and DsbB (probably involved in microcin-OmpF binding) (Zhao et al., 2015; Lu et al., 2019).

The gene *mtfI* encodes for MccN (Mcc24) immunity (O’Brien and Mahanty, 1994), and as in the previous 2 cases, it is also a protein with several transmembrane domains. MccN is closely related to MccE492, but lacking post-translational modifications (Corsini et al., 2010). The self-immunity phenotype is achieved in MccN producers by reducing the expression of the Mar operon regulator, MarR (multiple-antibiotic-resistance), which results in a phenotype resistance to other antimicrobial compounds (Carlson et al., 2001).

#### Microcins in *Enterobacteriaceae*

Inside *Enterobacteriaceae*, the production of microcins appears to be preferentially associated with some lineages. The first studies on microcins, using phenotypic methods, had estimated that 15% of *E*. *coli* strains isolated from newborns were microcin producers (Asensio et al., 1976). In fact, among the *Enterobacteriaceae* genomes of the publicly accessible National Centre for Biotechnology Information database, 34.1% of those corresponding to *E*. *coli* contain specific microcin gene sequences as defined in the APD3 (see text footnote 1) antimicrobial peptide database (Wang et al., 2016). Most of the various microcins are represented in *E*. *coli*, and are dominated by MccV (8.58%), MccM (7.43%), MccH47 (7.18%), and MccI47 (4.26%), with all other microcins at a frequency below 2%. Among the *Shigella sonnei* (closely related to *E*. *coli*) genomes examined, only 1.59% contained microcin genes; however, as in *E*. *coli*, a large variety of microcin genes were found, dominated by MccV (0.55%) and MccPDI (0.24%). *Shigella flexneri* is infrequently microcinogenic (0.54% of the strains). *S*. *enterica* has a low number of microcin producers (1.59%), and predominantly MccV (1.3%). *K*. *pneumoniae* harbors microcin genes in 4.65% of the strains, mostly MccE492 (4.19%), which was first discovered in this species. This microcin surprisingly was absent in *E*. *coli*; however, it was also scarcely represented (0.19%) in *Enterobacter cloacae*, a species with a low proportion of microcin producers (0.38%). Although *Citrobacter freundii* has 3.29% of microcinogenic strains, MccS was exclusively found, as in other *Citrobacter* species. However, MccS is the microcin more extensively distributed among Enterobacterial species. MccV was not found in either *Enterobacter* or *Citrobacter*. Another bioinformatic analysis has revealed that microcin C-like adenylated peptides are widespread and are encoded by both Gram-negative (including *Yersinia*) and Gram-positive bacteria, and even by cyanobacteria (Bantysh et al., 2014).

It is tempting to suggest that the high proportion of microcinogenic strains in *E*. *coli* and the diversity of microcins in this species, compared with other *Enterobacteriaceae*, correspond to a highly competitive lifestyle inside multiple intestinal subniches (microniches) in which even weak microcins can play a substantial ecological role (Majeed et al., 2013). Bacteria with fewer and more specific niches (including intracellularity), such as *Shigella* or *Salmonella*, are much less microcinogenic. In addition, *Klebsiella*, *Citrobacter*, and *Enterobacter*, which have a much broader environmental lifestyle (Sánchez-Valenzuela et al., 2017), have distinctive microcins that are rarely found in *E*. *coli*, even though the number of available genomic sequences is larger than that of other organisms. Strains of the environmental species *Serratia marcescens* might contain analogs to MccN (Gerc et al., 2014).

Inside *E*. *coli* species, other authors have applied bioinformatic methods for microcin gene searching. The phylogenetic classification of *E*. *coli* reflects macro-evolutionary events, bacterial sub-speciation-like processes that take place over long periods of time and space (Wirth et al., 2006; Turrientes et al., 2014). Respectively for the A, B1, B2, and D main *E*. *coli* phylogenetic lineages, MccV was found in 37, 29, 29, and 23% of the strains; MccM in 10, 12, 34, and 21%; MccB17 in 8, 10, 11, and 19%; MccC7 in 2, 2, 1, and 2%; and MccJ25 and MccL were only found in the B2 (1 and 1%, respectively) and D (2 and 1%, respectively) phylogroups. Thus, MccB17 was most common in phylogroup D, and MccV in phylogroup A (Micenková et al., 2016b).

The association between *E*. *coli* bacteremia and microcin production appears particularly solid in cases associated with urinary tract infections (Micenková et al., 2017). In the case of phylogroup B2, in which many high-risk clones are located, such as the globally widespread, highly invasive, and antibiotic-resistant clone O25B-ST131, the proportion of microcin producers duplicates the colicin-producing strains (Micenková et al., 2016a, b).

As stated earlier, microcins are not exclusive from *Enterobacteriaceae*. A genome-mining search in anaerobic bacteria has demonstrated that these quantitatively dominant populations of the gut microbiota also produce a significant proportion (approximately 25% in a heterogeneous sample of only 211 genomes) of RiPPs, frequently in conjunction with polyketides or non-ribosomal peptides (Letzel et al., 2014).

#### Ensembles of Microcins and Colicins

**FIGURE 2:**
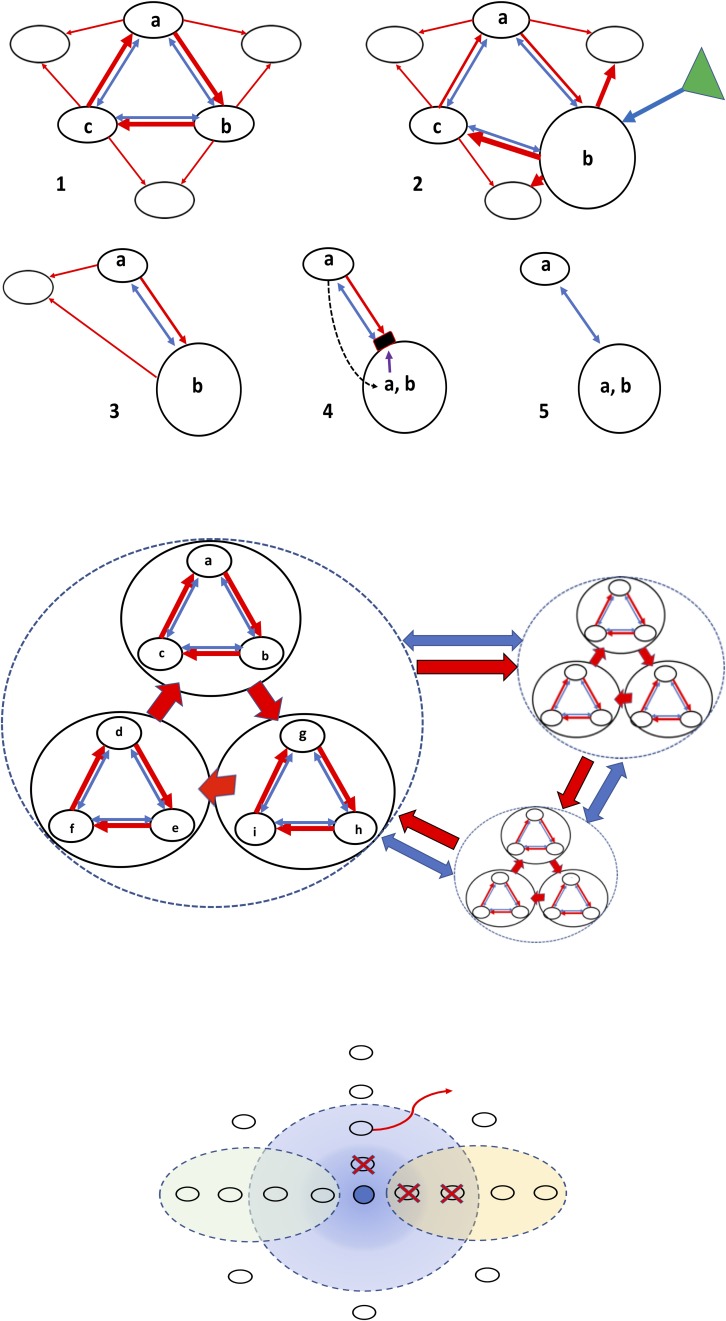
Structure of bacterial interactions and the influence of chemosphere. The structure and evolution of the microbiome is based in antagonistic and cooperative interactions in a complex chemical environment, the chemosphere. **Top panel**, antagonistic (red arrows) and cooperative (blue arrows) interactions among three bacterial populations producing different inhibitors (a–c). (1) The rock-paper-scissors dynamics, assuring coexistence of all three populations, which is enhanced by the cooperative blue bonds. This ensemble of populations cooperates in the inhibition of other competing bacteria (empty circles). (2) Under the influence of chemosphere (green triangle) one of the populations (b) increases in size, producing the collapse of the former equilibrium (3). In (4), because of the maintained coexistence with (a) and the high population size of (b), resistance to (a) might evolve in (b), or genes encoding (a) can be acquired by (b) via horizontal gene transfer, and a new, simpler coexistence might occur (5). **Middle panel**, the rock-paper-scissors dynamics at a higher hierarchical scale; ensembles of bacterial populations act as single entities able to compete and cooperate with other microbial ensembles. **Lower panel**, in the center, the dark blue circle represents a bacterial population excreting a “blue” microcin. The concentration of this bacteriocin is high near the producer, but diffusion gives rise to progressively lower concentrations (light blue). White circles, bacteria competing with the blue one, which (vertical line) are killed (red X) at high bacteriocin concentrations, or, at lower ones, prevented to be established (red curved arrow) in this area. In the left oval green circle, diffusion of a local chemosphere component antagonizing the production or effect of microcin, now unable to kill the competitors. In the yellow oval circle at the right, diffusion of a chemosphere component enhancing the effect of the bacteriocin, now able to kill even at very low concentrations.

Ensembles of inhibitory entities of microbial origin might exert stronger or broader spectrum inhibitory effects on competing organisms. On the other hand, these ensembles constitute a natural “combination strategy,” thus reducing the possibility of selection of single-entity mutants (a mutant resistant to one of the antibacterial compounds will probably be killed by the other one/s). Mutual killing assures biodiversity (Abrudan et al., 2012; Coyte et al., 2015). Finally, asymmetric ensembles could assure the permanence of natural species and clone diversity according to the previously mentioned rock-paper-scissors model (Czárán et al., 2002; Lenski and Riley, 2002; Kirkup and Riley, 2004; Reichenbach et al., 2007), and that might also occur at higher hierarchical levels, as for coexistence of small bacterial communities (Figure 2).

The formation of ensembles is certainly facilitated by horizontal gene transfer. Soon after the microcin discovery, the relevance of plasmids in microcin gene transfer was highlighted (Baquero et al., 1978). In fact, most microcins are plasmid-mediated; those of chromosomal location tend to be associated with genomic islands. For instance, the genes involved in the production and immunity of MccM and MccH47 in *E*. *coli* Nissle, 1917 are located in genomic island I, originated from horizontal genetic transfer (Grozdanov et al., 2004; Bravo-Vázquez, 2009). Microcins can be either chromosomally or plasmid encoded, whereas colicins have been found only on plasmids.

Interestingly, *E*. *coli* microcinogenic strains frequently express more than 1 microcin (even 4 in the same strain) (Sablé et al., 2003). Such coexistence might foster microcin evolution, including recombinatorial processes facilitated by the modular structure of some of these peptides. This has been suggested for MccV and MccH47, which might recombine genetic sequences corresponding to uptake and toxic modular domains (Azpiroz and Laviña, 2007). The frequent association of the microcins MccH47 and MccM in the same strain can also be favored by common systems of secretion and immunity.

Colicin-colicin, colicin-microcin, and microcin-microcin combinations were found to coincide in particular *E*. *coli* strains much more often than would be expected by chance. This combination occurs particularly in strains belonging to the phylogroup B2 and with associations between MccH47 and MccM; colicin Ia and MccV; colicins B and M; colicins E1 and M; and colicins E1 and Ia (Gordon and O’Brien, 2006). In colicinogenic enterohaemorrhagic strains, more than one-half of the strains produce multiple colicins, mostly B, E2/E7, and M (Schamberger and Díez-González, 2004). In general, as much as 40% of the *E*. *coli* strains in the intestine also show coexpression of colicins and microcins (Micenková et al., 2016a). For instance, the microcins MccH47 and MccM are produced by strains that are also producing colicin H (*E*. *coli* CA56) or colicin G (*E*. *coli* K58) (Patzer et al., 2003). Coexistence of genetic determinants of microcins and colicins in the same cell provides the opportunity for a recombinatorial exchange of fragments or eventually, the loss of one of these functions. The frequent colicins B and M are usually encoded adjacently on the same plasmid in *E*. *coli*; in some strains, this plasmid contains a remnant of the MccV operon next to a truncated colicin B activity gene, indicating recombination events between colicin BM and MccV plasmids (Christenson and Gordon, 2009). Moreover, the expression of a colicin (microcin?) from one producer can induce colicin production in a second producer and *vice versa* (Majeed et al., 2011). Secreted amino acid-based inhibitors, such as homoserine-transacetylase inhibitors (historical microcins Mcc93 and Mcc15) or peptidic microcins also coexist in the same strain (Aguilar et al., 1983).

In a previous section we detailed the mechanisms of resistance to microcins in non-microcin producers. Several mechanisms of microcin resistance, such as *E*. *coli* OmpF mutants, provide cross-resistance to other microcins, colicins, and even bacteriophages and antibiotics. In fact, there is a complex landscape in which resistance to each one of these entities might select for resistance to others. Another example is the mutants in FhuA, the *E*. *coli* outer membrane receptor for ferrichrome-iron (Destoumieux-Garzón et al., 2005). This protein also acts as the receptor for the phages T1, T5, UC-1, and f80 for colicin M (one of the smallest colicins, 29.4 kDa), and for the antibiotics albomycin, some rifamycins, and the microcin J25. Of course, mutations in common import mechanisms will produce resistance to all inhibitors using these systems; this resistance occurs with colicins and microcins using the active import Ton system (TonB, ExbB, and ExbD proteins), at the expense of energy provided by the proton motive force of the cytoplasmic membrane (Braun et al., 2002). Finally, although not addressed in this review, membrane-permeabilizing antimicrobial peptides present in the gut might sensitize bacterial cells to the effect of some microcins (Pomares et al., 2010).

#### The Ecological Significance of Microcins

The discovery of microcins was driven by the search for molecular mediators of bacterial displacements in the neonatal gut, and this ecological view was expressed very early in their study (de Lorenzo and Aguilar, 1984). The production of antimicrobial small antibiotic peptides by bacteria appears to be a widespread strategy in maintaining diversity in the intestinal microbiota. For example, bioinformatic detection of biosynthetic gene clusters in microbiota has revealed the numerical dominance of those involved in synthesis of the main microcin-related molecules, RiPPs (Arnison et al., 2013; Donia et al., 2014).

Microcins are eco-active molecules that are active in *Enterobacteriaceae* as mediators of inter- and intraspecies competition. An unintended natural experiment lasting for a century (1917–2019) has provided evidence for this assertion. An oral preparation of the strain *E*. *coli* Nissle, 1917 (Jacobi and Malfertheiner, 2011) has been used for over 100 years as a useful probiotic preparation for therapy of bacterial intestinal diseases, starting during the First World War (Nissle, 1916, 1918; Henker et al., 2007; Sonnenborn and Schulze, 2009). *E*. *col*i Nissle, 2017 has been also detected in swine herds, with similar protective effects against pathogens (Kleta et al., 2006). In the first years of the 21st century, it was discovered that *E*. *col*i Nissle 1917 produce 2 microcins, MccM and MccH47 (Patzer et al., 2003). A mutant *E*. *coli* Nissle 1917 strain unable to secrete these microcins was unable to outcompete other *E*. *coli* and *S*. *enterica* in the inflamed intestine, whereas the wild strain produced this ecological effect (Sassone-Corsi et al., 2016). Evidence for the ecological effects of other microcins are available, such as for MccV (Boubezari et al., 2018), the most abundant among the *E*. *coli* microcins (see above).

These results confirmed much earlier preliminary work on the effects of microcins on microbial gut interactions (Baquero and Asensio, 1979; Jorge, 1984). It would be expected that microcinogenic *E*. *coli* strains could reach higher population densities in the gut, facilitating translocation (and consequently bacteremia), and urinary tract infections. This association has already been reported (Azpiroz et al., 2009; Budič et al., 2011).

As previously stated, concerning microcin ecological functions in interbacterial interactions, microcins constitute defensive rather than attack molecules (Rebuffat, 2012). Most microcins are produced and excreted during a stationary phase, and are regulated by, for example, *rpoS* (RNA polymerase sigma factor), *ompR* (DNA-binding transcriptional dual regulator), and *spoT* (bifunctional (p)ppGpp synthase/hydrolase) (Moreno et al., 2002). These populations are, compared with invaders, of a higher population size, facilitating a sufficient local concentration of the inhibitor (Wiener, 1996). High cell density or local inflammation also increases competition for critical nutrients, such as iron, in such a way that favors the uptake of siderophore-microcins (Sassone-Corsi et al., 2016). In general, there is always a link between nutritional stress and competitive behavior in biology.

## Microcins in the Intestinal Chemosphere

In this review, the role of microcins as effectors of intermicrobial interactions has been highlighted. At the same time, we wanted to relativize the view of “single molecules” as the main characters of bacterial displacements, and in general, of microbial ecology. As previously stated, the microecological effects (microbe-microbe and microbe-environment) of individual cell metabolism might be critical in shaping the interactive dynamics and evolution of microbial ecosystems (Klitgord and Segrè, 2011). According to the postulate of “Molecular Ecology” (Asensio, 1976), any particular molecule inhibiting bacterial growth is necessarily surrounded by many others, which might have effects on the growth or inhibition of bacterial populations and/or in the production or stability of this molecule. The secretion of amino acids and amino acid derivatives with inhibitory activity might modify the uptake, export, and biosynthesis of bacterial peptides (Payne, 1977). Amino acid-based inhibitors (historically included as microcins) share with some peptidic microcins the “Trojan horse” strategy of mimicking essential nutrients (such as essential amino acids or iron-siderophores to penetrate inside the target cell).

### The Eco-Active Chemosphere

We have used the term “eco-active” to designate the part of the chemosphere constituted by chemicals able to play a role as factors of intestinal microbial ecology; i.e., growth-promoting molecules, growth-inhibiting (or killer) substances, and chemicals influencing bacterial genetic variation, genetic regulation, bacterial interactions, and colonization efficiency (Table 1). We are far from understanding in detail how this ensemble of chemical factors determines the microecological structure of the intestinal microbiota. A major limitation is the lack of knowledge about the spatial structure and organization of microenvironments (Baquero, 2015). It can be predicted that ensembles of bioactive molecules occur in microcompartments of the gut, which are dominated by particular microbial ensembles (Earle et al., 2015). How this constellation of molecules interacts and influences bacterial populations is an inconceivably complex issue. The possibility of intermolecular interactions between these molecules and with the microbiota depends on the “physics” and spatial dynamics of the system (intestinal ecophysics). For instance, the non-directional peristaltic movements of the colonic content assure complex mixing of bacterial populations (Ley et al., 2006) and of molecules from the chemosphere. However, bacterial populations in the gut probably interact at the microscopic scale inside clumps or aggregates, and these ensembles have their own chemospheres (Sonnenburg et al., 2005). In fact, specific chemospheres should be part of the “common niches” constructed by microbial consortia. However, all local chemospheres are open and fluctuating systems and are therefore influenced by the larger chemosphere in which they are embedded. Fluctuations in the chemosphere as a result of dietary changes and the host’s physiological, pathological, or therapeutic circumstances likely influence the complex microbiota. Combined cocausal effects are eventually able to influence the production, release, or the activity of growth-promoting and inhibitory substances. Not being part of the natural chemosphere, chemotherapeutic substances directly stressing the microbiota (such as antimicrobial agents), affecting the functionality of the intestine (such as drugs influencing peristalsis, cholagogues, or antacids) or influencing host immunity (such as corticoids, immunodepressive agents) could alter (stress) intestinal microecology and favor the bacterial expression of eco-active substances (Taymaz-Nikerel et al., 2013; Baquero, 2015; Gillis et al., 2018).

**TABLE 1:** Compounds in the intestinal chemosphere with antimicrobial effects, and their basic mechanisms of action.

**Polyphenols**	
• Quercetin	
• Chlorogenic acids	Bacterial membrane permeabilization

**Short-chain fatty acids**	
• Acetate	Lowering pH
• Propionate	pH-independent effects
• Butyrate	

**Organic salts**	
• Lactate	Lowering pH
• Citrate	pH-independent effects

**Bile acids, lipids**	
• Secondary bile acids and terpenoids	Disruption of cell membranes
• Short and medium-chain saturated fatty acids	Indirect effect: modulation of local innate immunity
• Long-chain unsaturated fatty acids	
• Fatty alcohols and fatty acid monoglycerides	
Polyaminated molecules	
• Spermidine, homospermidine, and norspermidine	Disruption of cell membranes
• Putrescine, cadaverine, and 1,3-diaminopropane	Regulation of bacteriocin production

**Intercellular signaling molecules**	
• Homoserine lactones	Bacterial membrane permeabilization
• Indole-based signaling molecules	Quorum-sensing signaling

**Haem catabolism**	
• Unconjugated bilirubin	Unknown, antioxidant effects?
• Biliverdin	
**Host defense secreted antimicrobials**	
•α-defensins, β-defensins, and cathelicidins	Antimicrobial peptides and disruption of cell membranes
• C-type lectins RNAses	Protective carbohydrate-bonding proteins Cytokine induction and endosomal pathways suppressing bacteria

**Immunoglobulins**	
• Secretory IgA	Capture bacterial cells (immune exclusion), facilitating immunological, and physical removal of bacteria
**Bacteriocins**	
• Colicins (class I–III)	Membrane pore formation and nuclease activity
• Historical amino acid-based microcins	Interference with amino acid metabolism
• Class I peptidic microcins (post-translational thiazole/oxazole-modified microcins)	Membrane pore formation, impairing cellular proton channel, protein synthesis inhibition, inhibition of DNA gyrase, inhibition of cellular respiration, plasmid post-segregational killing, and bacterial persistence phenotype
• Class IIa peptidic microcins	
• Class IIb peptidic microcins	

### Microcin Activity in Their Natural Chemosphere

Most of the studies published on the antimicrobial activity of microcins have been based on *in vitro* studies, in highly simplified environments. The intestinal chemosphere, variable in time and space, might determine particular configurations of interacting bacterial populations, and therefore the local effects of microcins are difficult to anticipate. *In vitro* models of the intestinal environment have been explored to predict the effect of microcins under gut conditions. For instance, MccJ25 was relatively stable under gastric conditions, but not in the duodenum conditions, being degraded by elastase I, and less efficiently α-chymotrypsin (Naimi et al., 2018). It has been shown that microcin inhibition does not occur in a rich nutrient system containing mucins or nucleic acids, as these molecules may bind peptides and suppress their antimicrobial activity (Ran et al., 2017). In the above section “The Intestinal Chemosphere and the Molecular Ecology of the Gut,” the basic compounds serving as bacterial nutrients in the chemosphere were considered. Changes in their absolute or relative concentrations should modify the growth rate and cell number of bacterial populations, and consequently their susceptibility to growth inhibitors or their ability to act as inhibitors of other populations (Figure 2). In Table 1 we summarize the main compounds in the intestinal chemosphere possessing antimicrobial activity. Certainly, the final effect of a microcin on a bacterial population depends on compounds facilitating bacterial growth, such as those acting as nutrients (reviewed in section “The Intestinal Chemosphere: Molecular Ecology”). Of particular importance are those nutrients that are critical but present at very low concentrations, which constitute important competition attractors, such as iron. In fact, siderophores frequently act in the internalization of microcins. Most importantly, microcins probably interact with many other bacterial inhibitors in the gut, either in a competitive or cooperative manner; however, this remains an almost unexplored field of research. The list of chemicals with inhibitory activity in Table 1 allows us to distinguish 2 main types of inhibitors. Many of them correspond to chemicals altering or disrupting bacterial membranes, likely increasing permeability to external compounds. A few (mostly microcins and colicins) have more specific modes of action, targeting cellular processes, such as protein synthesis or DNA replication, but also altering membrane integrity. We can easily conceive of a possible synergy between compounds altering cellular structures (membranes) and those inhibiting specific cellular processes, generally with higher intrinsic activity. This distinction has previously been considered by other authors, suggesting that bacteria from the gut seem to produce many bacteriocins with low activity and small number of highly effective bacteriocins (Drissi et al., 2015). Overall, the activity of microcins might be modulated by the chemosphere composition, which constitutes the main message of this review. Interventions to specifically modify the human and animal chemosphere will likely have important consequences in the epidemiology of normal and pathogenic microbiota, and in controlling antibiotic resistance (Baquero et al., 2013; Kashyap et al., 2013b). New developments in the study of the complex chemical microecology of the gut, the field of Asensio’s “Molecular Ecology” (Asensio, 1976), are certainly needed to obtain more realistic conclusions about the role of microcins in the interactive processes influencing the structure of microbiota.

## Author Contributions

FB and DB-V wrote the manuscript. All authors listed have made a substantial, direct and intellectual contribution to the work, and approved it for publication.

## Conflict of Interest

The authors declare that the research was conducted in the absence of any commercial or financial relationships that could be construed as a potential conflict of interest.
